# A Novel Intelligent System for Brain Tumor Diagnosis Based on a Composite Neutrosophic-Slantlet Transform Domain for Statistical Texture Feature Extraction

**DOI:** 10.1155/2020/8125392

**Published:** 2020-07-10

**Authors:** Shakhawan H. Wady, Raghad Z. Yousif, Harith R. Hasan

**Affiliations:** ^1^Applied Computer, College of Medicals and Applied Sciences, Charmo University, Chamchamal, Sulaimani, KRG, Iraq; ^2^Technical College of Informatics, Sulaimani Polytechnic University, Sulaimani, KRG, Iraq; ^3^Department of Information Technology, University College of Goizha, Sulaimani, KRG, Iraq; ^4^Department of Physics, College of Science, Salahaddin University, Erbil, KRG, Iraq; ^5^Department of IT, College of Information Technology, Catholic University in Erbil, KRG, Iraq; ^6^Department of Computer Science, Kurdistan Technical Institute, Sulaimani, KRG, Iraq; ^7^Computer Science Institute, Sulaimani Polytechnic University, Sulaimani, KRG, Iraq

## Abstract

Discrete wavelet transform (DWT) is often implemented by an iterative filter bank; hence, a lake of optimization of a discrete time basis is observed with respect to time localization for a constant number of zero moments. This paper discusses and presents an improved form of DWT for feature extraction, called Slantlet transform (SLT) along with neutrosophy, a generalization of fuzzy logic, which is a relatively new logic. Thus, a novel composite NS-SLT model has been suggested as a source to derive statistical texture features that used to identify the malignancy of brain tumor. The MR images in the neutrosophic domain are defined using three membership sets, true (*T*), false (*F*), and indeterminate (*I*); then, SLT was applied to each membership set. Three statistical measurement-based methods are used to extract texture features from images of brain MRI. One-way ANOVA has been applied as a method of reducing the number of extracted features for the classifiers; then, the extracted features are subsequently provided to the four neural network classification techniques, Support Vector Machine Neural Network (SVM-NN), Decision Tree Neural Network (DT-NN), *K*-Nearest Neighbor Neural Network (KNN-NN), and Naive Bayes Neural Networks (NB-NN), to predict the type of the brain tumor. Meanwhile, the performance of the proposed model is assessed by calculating average accuracy, precision, sensitivity, specificity, and Area Under the Curve (AUC) of the Receiver Operating Characteristic (ROC) curve. The experimental results demonstrate that the proposed approach is quite accurate and efficient for diagnosing brain tumors when the Gray Level Run Length Matrix (GLRLM) features derived from the composite NS-SLT technique is used.

## 1. Introduction

Most contemporary vision algorithms cannot accurately perform based on image intensity values which are directly derived from the initial gray level representation. Image intensity values are highly redundant, while the amount of important information within the image might be small. The Slantlet-based transformation of the initial MR image representation into a feature representation explicitly emphasizes the useful image features without losing essential image information, reduces the redundancy of the image data, and eliminates any irrelevant information [1]. Medical images perform a crucial role in disease analysis, education, investigation, etc. In the medical domain, due to the enormous development of digital medical images, an automated classification system of brain tumors is required to help radiologists accurately identify brain tumors or perform investigation based on brain Magnetic Resonance Imaging (MRI) [2, 3]. Since 2006, numerous systems were developed in the area of medical image, which relies mainly on the extraction of low-level features such as texture, intensity, shape, and color in order to understand, characterize, and classify medical images efficiently [2]. Medical image classification is a key issue in the field of image recognition, and it is intended to classify medical images into different categories. Basically, the classification of medical images can be divided into two phases of development. Effective image features are extracted from the first stage, and the second step is to use the features to construct an image dataset model [4]. Moreover, texture analysis, the mathematical method for quantitative analysis of image pattern variation, had shown promising diagnostic potential in different brain tumors that relate to an object's surface properties and its association with the adjacent region [5–7].

A brain tumor is one of the worst diseases that has risen due to an abnormal brain cell growth affecting the function of nervous systems. Various types of tumors in the brain may be benign or malignant. Cells of a benign brain tumor (low-grade glioma (LGG)) rarely invade healthy adjacent cells and have different boundaries and slow development of progression. Malignant brain tumor (HGG, BM, or recurrent glioma) cells readily invade brain or spinal cord neighboring cells and have fluid boundaries and rapid growth levels [8–10]. The early stage of tumor diagnosis relies on the doctor's knowledge and experience to help patients to recover and survive. An automated brain tumor classification system is an efficient tool to help physicians to successfully follow their treatment options [11, 12]. During the past years, several automatic methods for brain image analysis have been developed to detect and classify brain tumors using MR images.

The research paper [13] addresses a fully automated system for the identification of tumor slices and the delineation of the tumor region on the basis of two-dimensional anatomic MR images. Features were extracted using Gabor wavelet and statistical feature extraction techniques, and they achieved the highest classification result with statistical features in comparison to Gabor wavelet features. Subashini and Gandhi [14] and his coworkers published an article on automatic detection and classification of MRI brain tumors using LabVIEW. A dataset of 80 images was utilized to test this approach, and they achieved 92.5% of classification accuracy. In another work [15], the authors proposed a 2-level DWT method to extract features from MR images. In the method, feature selection using PCA and DNN models was used for brain MRI classification into normal and three categories of malignant brain tumors. Gupta et al. [16] proposed a noninvasive system for brain glioma detection on brain MRIs using texture and morphological features with ensemble learning. Simulations were scored 97.37% and 98.38 on JMCD and BraTS, respectively. In [17], the authors developed a clinical support system to enhance brain tumor detection and classification using images from the BraTS dataset. The tumor region's features were collected by the GLCM extraction technique and classified using LOBSVM with 97.69% accuracy. An approach of a deep learning (DL) model based on a CNN for the classification of brain tumor MR images was suggested by Sultan et al. [18]. The proposed system attained a substantial performance with the best overall accuracy of 98.7%. In Reference [18], the authors have addressed the new liver and brain tumor classification approach using CNN, DWT, and LSTM for feature extraction, signal processing, and signal classification, respectively. Experimental results showed that hybrid CNN-DWT-LSTM algorithms were substantially better performing, and they achieved overall performance of 98.6%. In 2019, Ullah et al. [19] developed a modified scheme to differentiate between normal and abnormal brain MR images based on a median filter, DWT, color moments, and ANN. In [20], the author proposed a machine learning approach based on delta-radiomic features of DSC-MR images. The developed algorithm was used for classifying HG and LG GBMs with an average of 90% accuracy.

Over the past few decades, many methods have been proposed in the literature for feature extraction. These techniques were based on features extracted from spatial and frequency domains, and it was observed that very few studies have been conducted on brain tumor diagnosis based on the neutrosophic domain. Amin and his colleagues [21] developed a new system of neutrosophic ranking for classifying tumors in BUS images. In the system, original BUS images were transformed into a neutrosophic set domain and various features were extracted from statistical and morphological features. Sert and Avci [22] proposed a neutrosophic set EMFSE system using maximum fuzzy entropy and fuzzy c-partition methods to identify the enhancing part of the tumor in a brain MR image. The authors in [23] proposed an effective automatic brain tumor segmentation scheme based on the NS-EMFSE method for classifying brain tumors as benign and malignant with the SVM and KNN classifier. A dataset of 500 samples was taken from various cancer categories for the TCGA-GBM dataset to test this approach, and they achieved the highest performance by the SVM classifier with 95.62%.

### 1.1. Neutrosophy

Neutrosophy is a branch of philosophy, introduced by F. Smarandache in 1980, which generalized dialectics and studied the origin, nature, and scope of neutralities, in addition to their interactions with numerous ideational spectra [24]. In neutrosophy theory, every event has a definite degree of truth (*T*), falsity (*F*), and indeterminacy (*I*) that have to be considered independently from each other [23, 25–28]. Therefore, {*A*} is an idea, theory, event, concept, or entity; {Anti − *A*} is the opposite of {*A*}; and the neutrality {Neut − *A*} means neither {*A*} nor {Anti − *A*}, that is, the neutrality between the two extremes [29, 30].

### 1.2. Concept of Neutrosophic Set

**Figure 1:**
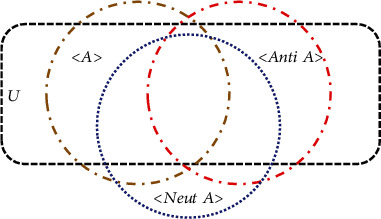
Neutrosophic diagram.

A neutrosophic set is a generalization of the theory of fuzzy set, intuitionistic fuzzy set, paraconsistent set, dialetheist set, paradox set, and tautological set where each element of the universe has a degree of truth, falsity, and indeterminacy, respectively. Unlike in fuzzy sets, the neutrosophic set presents the additional domain (*I*) which provides a more effective way to handle higher degrees of uncertainty. Let *U* be a universe of discourse set and a neutrosophic set *A* in *U* is characterized by three neutrosophic components: *T*, *F*, and *I* are defined to estimate the membership degree (truth membership degree), nonmembership degree (falsity membership degree), and the indeterminacy membership degree of an element independently. The neutrosophic schema in the general case is shown in Figure 1.

The novelty of the proposed approach is to apply Slantlet transform in each of the neutrosophic sets to extract statistical texture features, which has not been explored and performed on MICCAI BraTS dataset. Furthermore, different individual and combined feature extraction methods using composite NS-SLT were compared through their classification accuracies to select the effective approach with four types of neural network classification techniques. To evaluate the performance, extensive experiments were carried out which show that the proposed composite system achieves excellent results and classifies images accurately.

## 2. Materials and Methods

**Figure 2:**
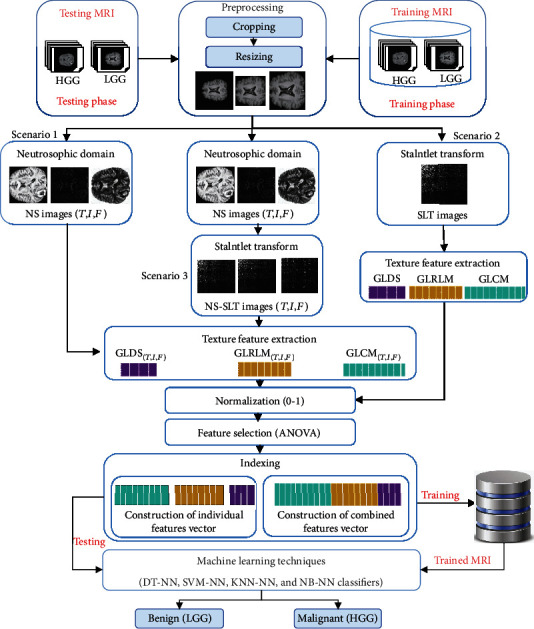
General architecture of the proposed system.

The overall design of the proposed framework is shown in Figure 2. First, MR images of patients are acquired, cropped, and resized in the preprocessing step; then, statistical texture features are extracted from SLT in the neutrosophic domain. Afterwards, feature selection is performed to choose the most salient features, followed by applying four neural network classifiers to identify the tumor as benign or malignant derived from the extracted features. Finally, the performance is evaluated by using certain parameters. The detail of these given methods has been presented in the subsequent subsections.

### 2.1. Dataset

**Figure 3:**
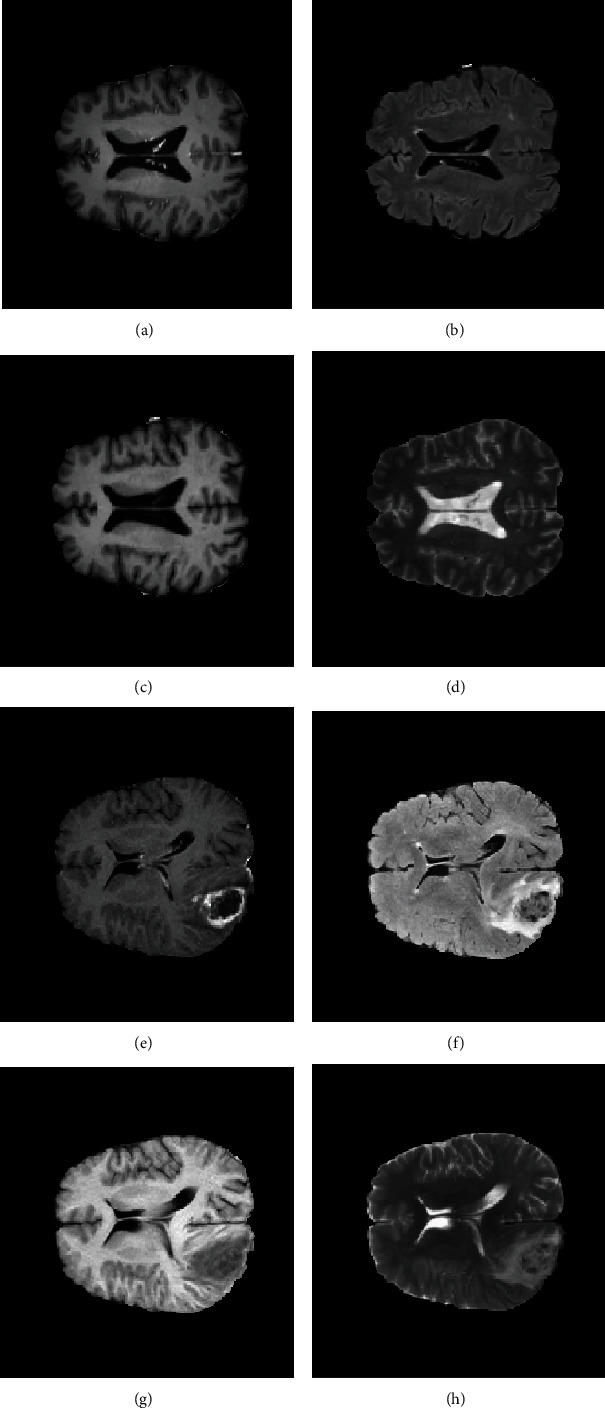
Samples from dataset for each class of brain tumors: (a) T1Ce benign image, (b) FLAIR benign image, (c) T1 benign image, (d) T2 benign image, (e) T1Ce malignant image, (f) FLAIR malignant image, (g) T1 malignant image, and (h) T2 malignant image.

Images in the MICCAI Brain Tumor Segmentation 2017 Challenge (BraTS 2017) were used to analyze and evaluate our proposed approach, which is one of the standard and benchmarked datasets [9, 31–33]. It is comprised of 210 preoperative MR images of patients from high-grade glioma (HGG) volumes and 75 MRIs from low-grade glioma (LGG) volumes collected from multiple centers. For each patient, there are four MRI modalities, including the native T1-weighted (T1), contrast-enhanced T1-weighted (T1ce), T2-weighted (T2), and T2 fluid-attenuated inversion recovery (FLAIR) (Figure 3). After their preprocessing, the data provided are distributed, i.e., skull-stripped, coregistered to the same anatomical template, and with the same resolution interpolated into 1 × 1 × 1 mm^3^ and with a sequence size of 240 × 240 × 155. In order to homogenize data, each modality scan is rigidly coregistered with T1Ce modality, because in most cases, T1Ce has the highest spatial resolution. Therefore, for our experiments, 285 brain MRI tumor (T1Ce) images are used, out of which 210 were cancerous (malignant) tumors from HGG and 75 were benign tumors from LGG.

### 2.2. Preprocessing

In the preprocessing stage, the input images (axial images) were initialized. The middle slice in an MRI volume is considered to have all the tissue regions. The pixels (nonobject) in the background are usually very prominent in MR images, and the processing time of brain extraction can be reduced considerably by separating target pixels from background pixels. Therefore, in this step, the bounding box cropping approach is computed in order to extract the brain portion alone as the AOI by removing the unwanted background from the input image. Before importing the input MR images into the system, the cropped MR images are resized into 512∗512 pixels.

### 2.3. The Image in Neutrosophic Domain

**Figure 4:**
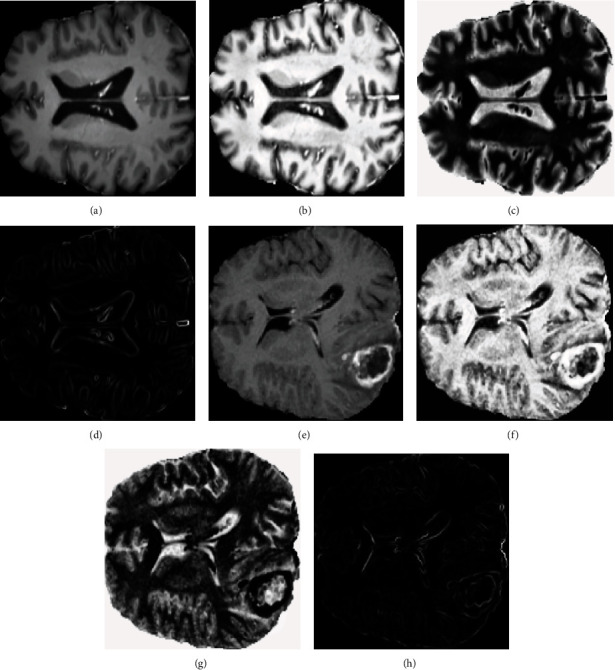
Samples from each class of brain tumors in the neutrosophic domain: (a) original image (benign), (b) *T* domain of benign image, (c) *F* domain of benign image, (d) *I* domain of benign image, (e) original image (malignant), (f) *T* domain of malignant image, (g) *F* domain of malignant image, and (h) *I* domain of malignant image.

Let *U* be a universe of discourse and *A* be a set included in *U*, which is composed of bright pixels. The image in the neutrosophic domain (*P*_NS_) is represented using three distinctive membership components (*T*, *I*, and *F*), where *T* defines the truth scale, *F* defines the scale of false, and *I* characterizes the scale of intermediate. All considered components are autonomous from each other. A pixel (*P*) of an image in the neutrosophic domain is characterized as *P*(*T*, *I*, *F*) [26–28, 30, 34] and belongs to set *A* in the following way: it is *t*% true membership function in the bright pixel set, *i*% indeterminacy membership function in the set, and *f*% a falsity-membership function in the set, where *t* varies in *T*, *i* varies in *I*, and *f* varies in *F*. There is a valuation for each component in [0, 1]. In the image domain, pixel *P*(*i*, *j*) is transformed into a neutrosophic domain by calculating *P*_NS_(*i*, *j*) = {*T* (*i*, *j*), *I* (*i*, *j*), *F* (*i*, *j*)} in equations (1), (2), (3), (4), (5) and (6), where *T*(*i*, *j*), *I*(*i*, *j*), and *F*(*i*, *j*) considered as a probability that pixel *P*(*i*, *j*) belongs to white set (object), indeterminate set, and nonwhite set (background), respectively (see Figure 4). This is the primary benefit of neutrosophy in image processing, and it can be taken at the same time when the decision is made for each pixel in the image. In [22, 23, 35–38], the following basic equations were proposed for transforming images from a pixel domain to the neutrosophic domain:

(1) $$\begin{array}{c} P_{\text{NS}}\left(i,j\right)=\left\{T\;\left(i,j\right),I\;\left(i,j\right),F\;\left(i,j\right)\right\}, \end{array}$$

(2) $$\begin{array}{c} T\left(i,j\right)=\frac{{\bar{g}}_{\left(i,j\right)}-{\bar{g}}_{\min}}{{\bar{g}}_{\max}-{\bar{g}}_{\min}}, \end{array}$$

(3) $$\begin{array}{c} {\bar{g}}_{\left(i,j\right)}=\frac{1}{A^{2}}\overset{i+a/2}{\underset{m=i-a/2}{\sum }}\overset{j+a/2}{\underset{n=j-a/2}{\sum }}g_{\left(m,n\right)}, \end{array}$$

(4) $$\begin{array}{c} I\left(i,j\right)=\frac{\delta_{\left(i,j\right)}-\delta_{\min}}{\delta_{\max}-\delta_{\min}\;}, \end{array}$$

(5) $$\begin{array}{c} \delta_{\left(i,j\right)}=\left|\;g_{\left(i,j\right)}-{\bar{g}}_{\left(i,j\right)}\right|, \end{array}$$

(6) $$\begin{array}{c} F\left(i,j\right)=1-T\;\left(i,j\right), \end{array}$$

where *g*_(*i*, *j*)_ represents the intensity value of an image in the pixel domain; *T*, *I*, and *F* are true, indeterminacy, and false sets, respectively, in the neutrosophic domain; ${\bar{g}}_{\left(i,j\right)}$ can be defined as the local mean value of *g*_(*i*, *j*)_; and *δ*_(*i*, *j*)_ is the homogeneity value of *T* at (*i*, *j*), which is described by the absolute value of the difference between intensity value of an image *g*_(*i*, *j*)_ and its local mean value ${\bar{g}}_{\left(i,j\right)}$.

### 2.4. Slantlet Transform (SLT)

The Slantlet transform is an improved orthogonal DWT variant with two zero moments and better time localization which was first utilized by Selesnick to evaluate nonstationary signals [39]. DWT is usually carried out by filter bank iteration, where a tree structure is utilized. Slantlet transform is inspired by an equivalent DWT implementation, in which a filter bank in a parallel structure is implemented [40]. DWT utilizes a product form of basic filters in some of these parallel branches, and the filter bank “Slantlet” uses a similar structure in parallel. However, there is no product type of implementation for the component filter branches, which means that SLT has extra independence. SLT will produce a filter bank, where each filter has its length in the power of 2; this results in a periodic output for the analysis filter bank and reduces the samples (2*i*–2) which support approaches one-thirds, as (*i*) increases [41].

**Figure 5:**
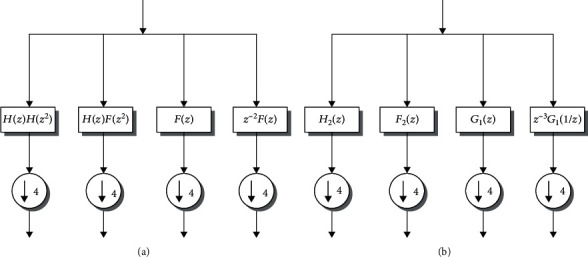
The two-scale iterated D2 filter bank (a) and two-scale SLT filter bank (b) [40].

For a mathematical perspective of the transformation of Slantlet, let us take a generalized representation of Figure 5, for (*l*) scales. The filters in scale (*i*) must be *g*_*i*_(*n*), *f*_*i*_(*n*), and *h*_*i*_(*n*) to analyze the signal where each filter has an appropriate 2^*i*+1^ support. For (*l*), the SLT filter bank uses (*l*) number of pairs of channels, i.e., (2*l*) channels in total. The low pass *h*_*i*_(*n*) filter is then combined with its adjacent *f*_*i*_(*n*) filter, where a downsampling of 2^*i*^ is followed by any filter. The channel pairs of each (*l* − 1) constitute a *g*_*i*_(*n*), followed by a downsampling by 2^*i*+1^ and the downsample by a reversed time version *i* = 1, 2, 3, ⋯, *l* − 1. The following expressions are represented by the following, as the filters *g*_*i*_(*n*), *f*_*i*_(*n*), and *h*_*i*_(*n*) implement linear forms in pieces:

(7) $$\begin{array}{c} g_{i}\left(n\right)=\left\{\begin{array}{cc} a_{0,0}+a_{0,1}n, & \text{for }n=0,\cdots ,2^{i}-1\\ a_{1,0}+a_{1,1}n, & \text{for }n=2^{i},\cdots ,2^{i+1}-1 \end{array}\right\},\\ h_{i}\left(n\right)=\left\{\begin{array}{cc} b_{0,0}+b_{0,1}n, & \text{for }n=0,\cdots ,2^{i}-1\\ b_{1,0}+b_{1,1}n, & \text{for }n=2^{i},\cdots ,2^{i+1}-1 \end{array}\right\},\\ f_{i}\left(n\right)=\left\{\begin{array}{cc} c_{0,0}+c_{0,1}n, & \text{for }n=0,\cdots ,2^{i}-1\\ c_{1,0}+c_{1,1}n, & \text{for }n=2^{i},\cdots ,2^{i+1}-1 \end{array}\right\}. \end{array}$$

**Figure 6:**
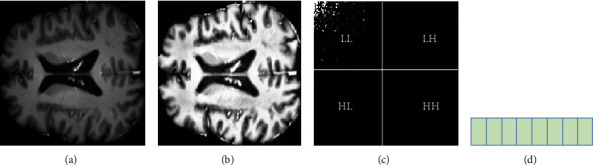
Samples from brain tumors: (a) preprocessed image, (b) image in NS domain (*T* image), (c) Slantlet transform image, and (d) extracted feature vector.

Two issues must be taken into account when computing SLT on MR images. Firstly, input signal length should be power of two, or higher than, the analysis filter bank length of the SLT, since all filter lengths are power of two in SLT filter bank. Secondly, the matrix of transformation has to be constructed. In a 2D SLT decomposition, there is usually an image that is divided into two parts: approximation and detailed parts. The approximation part includes one low-frequency LL subband, and detailed parts include three high-frequency subbands: LH, HL, and HH, as Figure 6 illustrates, where H and L represent the high- and low-frequency bands, respectively. The low-frequency subband component (LL) includes the inventive information of the original image. On the contrary, the LH, HL, and HH subbands retain the information associated with the contour, edge, and the image's other details. In the image, high coefficients characterize the important information; the low (insignificant) coefficients meanwhile are deliberated as trivial information or noise. Therefore, such small coefficients should be avoided for the best results. In this work, the SLT was utilized on MR images in spatial and neutrosophic domains to extract the statistical features of the images.

### 2.5. Feature Extraction

Feature extraction is the process of transforming the raw pixel values from an image into a set of features, normally distinctive properties of input patterns that can be used in the selection and classification tasks. Feature extraction techniques are usually divided into the geometrical, statistical, model-based, and signal processing [14, 16, 18, 42]. This stage involves obtaining important features extracted from MR images. The main features can be used to indicate the texture property, and the information is stored in the knowledge base for the system training. Three sets of statistical texture features (GLDS, GLRLM, and GLCM) are included for feature extraction in the proposed system. The obtained texture features by different methods are used individually and fused with each other for the classification process.  Table 1 shows all 22 statistical textural features extracted from each technique.

#### 2.5.1. Gray Level Cooccurrence Matrix (GLCM)

GLCM is one of the most widespread techniques of texture analysis that quantitatively measured the frequency of different combinations of pixel brightness values (gray levels) which occur in an image, and it has been used in a number of applications, e.g., [42–48]. In this step, texture features that contain information about the image are computed by GLCM to extract second-order statistic texture features (Table 1).

*(1) Neutrosophic Image Homogeneity*. Homogeneity also called inverse difference moment is a value that measures the similarity of the distribution of elements in the gray level cooccurrence matrix which is defined in [48]. The values vary between 0 and 1, and a higher value reveals a smoother texture feature.

Mathematically, homogeneity of an image in the spatial domain is defined as

(8) $$\begin{array}{c} \text{Homogeneity}=\overset{N-1}{\underset{i=0}{\sum }}\overset{N-1}{\underset{j=0}{\sum }}\frac{1}{1+{\left(i-j\right)}^{2}}\cdot P\left(i,j\right), \end{array}$$

where *P*(*i*, *j*) denotes element *i*, *j* of GLCM; *N* is the number of gray levels in the image; and *i*, *j* demonstrates the number of rows and columns in the image.

The neutrosophic image homogeneity is defined as the summation of the homogeneities of three sets *T*, *I*, and *F*. The basic equations to transform images from the pixel domain to the neutrosophic domain are calculated as follows:

(9) $$\begin{array}{c} {\text{NS}}_{\text{Homogeneity}}={\text{HOM}}_{\left(T\right)}+{\text{HOM}}_{\left(I\right)}+{\text{HOM}}_{\left(F\right)},\\ {\text{HOM}}_{\left(T\right)}=\overset{N-1}{\underset{i=0}{\sum }}\overset{N-1}{\underset{j=0}{\sum }}\frac{1}{1+{\left(i-j\right)}^{2}}\cdot P_{T}\left(i,j\right),\\ {\text{HOM}}_{\left(I\right)}=\overset{N-1}{\underset{i=0}{\sum }}\overset{N-1}{\underset{j=0}{\sum }}\frac{1}{1+{\left(i-j\right)}^{2}}\cdot P_{I}\left(i,j\right),\\ {\text{HOM}}_{\left(F\right)}=\overset{N-1}{\underset{i=0}{\sum }}\overset{N-1}{\underset{j=0}{\sum }}\frac{1}{1+{\left(i-j\right)}^{2}}\cdot P_{F}\left(i,j\right). \end{array}$$

*(2) Neutrosophic Image Energy*.

(10) $$\begin{array}{c} \text{ENR}=\overset{N-1}{\underset{i=0}{\sum }}\overset{N-1}{\underset{j=0}{\sum }}\left(P{\left(i,j\right)}^{2}\right),\\ {\text{NS}}_{\text{Energy}}={\text{ENR}}_{\left(T\right)}+{\text{ENR}}_{\left(I\right)}+{\text{ENR}}_{\left(F\right)}. \end{array}$$

*(3) Neutrosophic Image Entropy*.

(11) $$\begin{array}{c} \text{ENT}=-\overset{N-1}{\underset{i=0}{\sum }}\overset{N-1}{\underset{j=0}{\sum }}P\left(i,j\right)\cdot \log\left[P\left(i,j\right)\right],\\ {\text{NS}}_{\text{Energy}}={\text{ENR}}_{\left(T\right)}+{\text{ENR}}_{\left(I\right)}+{\text{ENR}}_{\left(F\right)}. \end{array}$$

*(4) Neutrosophic Image Contrast*.

(12) $$\begin{array}{c} \text{CON}=\overset{N-1}{\underset{n=0}{\sum }}n^{2}\overset{N-1}{\underset{i=0}{\sum }}\overset{N-1}{\underset{j=0}{\sum }}P\left(i,j\right),\;n=\left|i-j\right|,\\ {\text{NS}}_{\text{Contrast}}={\text{CON}}_{\left(T\right)}+{\text{CON}}_{\left(I\right)}+{\text{CON}}_{\left(F\right)}. \end{array}$$

*(5) Neutrosophic Image Symmetry*.

(13) $$\begin{array}{c} \text{SYM}=\overset{N-1}{\underset{i=0}{\sum }}\overset{N-1}{\underset{j=0}{\sum }}\left|P\left(i,j\right)-P\left(j,i\right)\right|,\\ {\text{NS}}_{\text{Symmetry}}={\text{SYM}}_{\left(T\right)}+{\text{SYM}}_{\left(I\right)}+{\text{SYM}}_{\left(F\right)}. \end{array}$$

*(6) Neutrosophic Image Correlation*.

(14) $$\begin{array}{c} \text{COR}=\overset{N-1}{\underset{i=0}{\sum }}\overset{N-1}{\underset{j=0}{\sum }}\frac{\left(i,j\right)\cdot P\left(i,j\right)-\left(\mu_{x}\cdot \mu_{y}\right)}{\left(\sigma_{x}\cdot \sigma_{y}\right)},\\ {\text{NS}}_{\text{Correlation}}={\text{COR}}_{\left(T\right)}+{\text{COR}}_{\left(I\right)}+{\text{COR}}_{\left(F\right)}. \end{array}$$

*(7) Neutrosophic Image Moment 1*.

(15) $$\begin{array}{c} {\text{MOM}}^{1}=\overset{N-1}{\underset{i=0}{\sum }}\overset{N-1}{\underset{j=0}{\sum }}\left(i-j\right)\cdot P\left(i,j\right),\\ {\text{NS}}_{{\text{Moment}}^{1}}={\text{MOM}}_{\left(T\right)}^{1}+{\text{MOM}}_{\left(I\right)}^{1}+{\text{MOM}}_{\left(\text{F}\right)}^{1}. \end{array}$$

*(8) Neutrosophic Image Moment 2*.

(16) $$\begin{array}{c} {\text{MOM}}^{2}=\overset{N-1}{\underset{i=0}{\sum }}\overset{N-1}{\underset{j=0}{\sum }}{\left(i-j\right)}^{2}\cdot P\left(i,j\right),\\ {\text{NS}}_{{\text{Moment}}^{2}}={\text{MOM}}_{\left(T\right)}^{2}+{\text{MOM}}_{\left(I\right)}^{2}+{\text{MOM}}_{\left(F\right)}^{2}. \end{array}$$

*(9) Neutrosophic Image Moment 3*.

(17) $$\begin{array}{c} {\text{MOM}}^{3}=\overset{N-1}{\underset{i=0}{\sum }}\overset{N-1}{\underset{j=0}{\sum }}{\left(i-j\right)}^{3}\cdot P\left(i,j\right),\\ {\text{NS}}_{{\text{Moment}}^{3}}={\text{MOM}}_{\left(T\right)}^{3}+{\text{MOM}}_{\left(I\right)}^{3}+{\text{MOM}}_{\left(F\right)}^{3}. \end{array}$$

*(10) Neutrosophic Image Moment 4*.

(18) $$\begin{array}{c} {\text{MOM}}^{4}=\overset{N-1}{\underset{i=0}{\sum }}\overset{N-1}{\underset{j=0}{\sum }}{\left(i-j\right)}^{4}\cdot P\left(i,j\right),\\ {\text{NS}}_{{\text{Moment}}^{4}}={\text{MOM}}_{\left(T\right)}^{4}+{\text{MOM}}_{\left(I\right)}^{4}+{\text{MOM}}_{\left(F\right)}^{4}. \end{array}$$

#### 2.5.2. Gray Level Run Length Matrix (GLRLM)

The concept, GRLM, is based on the reality that many neighboring pixels with the same gray level are characterized by coarse texture features [42, 44, 45, 47]. For a given image, GLRLM *P*(*i*, *j*) is calculated by representing the total runs of pixels having gray level *i* and run length *j* in a particular direction. Textural features are calculated from a set of components used to explore the essence of the textures of the image. Many numerical texture measurements can be calculated from the original run-length matrix *P*(*i*, *j*). At the end, eight original features of run length statistics for the neutrosophic domain are derived (Table 1).

*(1) Neutrosophic Image Short Run Emphasis (SRE)*.

(19) $$\begin{array}{c} \text{SRE}=\frac{1}{N_{r}}\overset{M-1}{\underset{i=0}{\sum }}\overset{N-1}{\underset{j=0\;}{\sum }}\frac{P\left(i,j\right)}{j^{2}}, \end{array}$$

where *P*(*i*, *j*) denotes the number of runs of pixels that have gray level *i* and length group *j*; *N*_*r*_ is the total number of runs in the image; *M* is the number of gray levels (bins); and *N* is the number of run lengths (bins):

(20) $$\begin{array}{c} {\text{NS}}_{\text{SRE}}={\text{SRE}}_{\left(T\right)}+{\text{SRE}}_{\left(I\right)}+{\text{SRE}}_{\left(F\right)}. \end{array}$$

*(2) Neutrosophic Image Long Run Emphasis (LRE)*.

(21) $$\begin{array}{c} \text{LRE}=\frac{1}{N_{r}}\overset{M-1}{\underset{i=0}{\sum }}\overset{N-1}{\underset{j=0}{\sum }}P\left(i,j\right)\cdot j^{2},\\ {\text{NS}}_{\text{LRE}}={\text{LRE}}_{\left(T\right)}+{\text{LRE}}_{\left(I\right)}+{\text{LRE}}_{\left(F\right)}. \end{array}$$

*(3) Neutrosophic Image Gray Level Nonuniformity (GLN)*.

(22) $$\begin{array}{c} \text{GLN}=\frac{1}{N_{r}}\;\overset{M-1}{\underset{I=0}{\sum }}{\left(\overset{N-1}{\underset{J=0}{\sum }}P\left(i,j\right)\right)}^{2},\\ {\text{NS}}_{\text{GLN}}={\text{GLN}}_{\left(T\right)}+{\text{GLN}}_{\left(I\right)}+{\text{GLN}}_{\left(F\right)}. \end{array}$$

*(4) Neutrosophic Image Run Percentage (RP)*.

(23) $$\begin{array}{c} \text{RP}=\frac{N_{r}}{N_{p}}, \end{array}$$

where *N*_*p*_ is the total number of pixels in the image:

(24) $$\begin{array}{c} {\text{NS}}_{\text{RP}}={\text{RP}}_{\left(T\right)}+{\text{RP}}_{\left(I\right)}+{\text{RP}}_{\left(F\right)}. \end{array}$$

*(5) Neutrosophic Image Run Length Nonuniformity (RLN)*.

(25) $$\begin{array}{c} \text{RLN}=\frac{1}{N_{r}}\;\overset{N-1}{\underset{j=0}{\sum }}{\left(\overset{M-1}{\underset{i=0}{\sum }}P\left(i,j\right)\right)}^{2},\\ {\text{NS}}_{\text{RLN}}={\text{RLN}}_{\left(T\right)}+{\text{RLN}}_{\left(I\right)}+{\text{RLN}}_{\left(F\right)}. \end{array}$$

*(6) Neutrosophic Image Low Gray Level Run Emphasis (LGRE)*.

(26) $$\begin{array}{c} \text{LGRE}=\frac{1}{N_{r}}\overset{M-1}{\underset{i=0}{\sum }}\overset{N-1}{\underset{j=0}{\sum }}\frac{P\left(i,j\right)}{i^{2}},\\ {\text{NS}}_{\text{LGRE}}={\text{LGRE}}_{\left(T\right)}+{\text{LGRE}}_{\left(I\right)}+{\text{LGRE}}_{\left(F\right)}. \end{array}$$

*(7) Neutrosophic Image High Gray Level Run Emphasis (HGRE)*.

(27) $$\begin{array}{c} \text{HGRE}=\frac{1}{N_{r}}\overset{M-1}{\underset{i=0}{\sum }}\overset{N-1}{\underset{j=0\;}{\sum }}P\left(i,j\right)\cdot i^{2},\\ {\text{NS}}_{\text{HGRE}}={\text{HGRE}}_{\left(T\right)}+{\text{HGRE}}_{\left(I\right)}+{\text{HGRE}}_{\left(F\right)}. \end{array}$$

#### 2.5.3. Gray Level Difference Statistics (GLDS)

The GLDS emphasizes the histogram of the absolute differences in the gray level between the two pixels that are separated by a displacement vector to calculate the tumor region's texture coarseness [49]. Let *d* = (*dx*, *dy*) be the displacement vector between two image pixels and *g*(*d*) the gray level difference at distance (*d*):

(28) $$\begin{array}{c} g\left(d\right)=\left|\;f\left(i,j\right)-f\left(i+dx,j+dy\right)\right|. \end{array}$$

*P*_*g*_(*g*, *d*) is the histogram of the gray level differences at the specific distance (*d*). One distinct histogram exists for each distance *d*. The following four statistical features were derived from the histogram of gray level differences in the neutrosophic domain (Table 1).

*(1) Neutrosophic Image Angular Second Moment*.

(29) $$\begin{array}{c} \text{ASM}=\overset{M}{\underset{i=1}{\sum }}{\left(P_{g}\left(\;g_{i},d\;\right)\right)}^{2},\\ {\text{NS}}_{\text{MEN}}={\text{ASM}}_{\left(T\right)}+{\text{ASM}}_{\left(I\right)}+{\text{ASM}}_{\left(F\right)}. \end{array}$$

*(2) Neutrosophic Image Contrast*.

(30) $$\begin{array}{c} \text{CON}=\overset{M}{\underset{i=1}{\sum }}g_{i}^{2}P_{g}\left(g_{i},d\right),\\ {\text{NS}}_{\text{MEN}}={\text{CON}}_{\left(T\right)}+{\text{CON}}_{\left(I\right)}+{\text{CON}}_{\left(F\right)}. \end{array}$$

*(3) Neutrosophic Image Mean*.

(31) $$\begin{array}{c} \text{MEN}=\overset{M}{\underset{i=1}{\sum }}g_{i}P_{g}\left(g_{i},d\right),\\ {\text{NS}}_{\text{MEN}}={\text{MEN}}_{\left(T\right)}+{\text{MEN}}_{\left(I\right)}+{\text{MEN}}_{\left(F\right)}. \end{array}$$

*(4) Neutrosophic Image Entropy*.

(32) $$\begin{array}{c} \text{ENT}=-\overset{M}{\underset{i=1}{\sum }}P_{g}\left(g_{i},d\right)\cdot \ln P_{g}\left(g_{i},d\right),\\ {\text{NS}}_{\text{ENT}}={\text{ENT}}_{\left(T\right)}+{\text{ENT}}_{\left(I\right)}+{\text{ENT}}_{\left(F\right)}. \end{array}$$

### 2.6. Feature Selection

The large number of texture features causes difficulty in ranking, prolongs computational time, and involves more memory space. Thus, the selection of features was regarded as part of the design of the proposed system. In our paper, the analysis of variance (ANOVA) technique was used to reduce the dimension of data based on its significance and variance and avoid losing too much information (Table 2). ANOVA is a powerful tool for determining if two or more sets of data have a statistically significant difference [50]. A normalization process on the input feature set was performed as part of data preparation prior to applying the ANOVA method.

### 2.7. Classification of Brain Tumors

Classification is a machine learning technique in which training data are used for building models and the model is used to predict new data [9, 16, 21, 51, 52]. In order to evaluate algorithm performance, the developed model is evaluated using testing data. Classification includes a wide range of decision-making approaches that are used in the CAD system [4]. Pixel-based image classification techniques analyze the numerical properties of selected image feature vectors and organize data into categories. In this study, four different classification techniques have been used, namely, DT-NN, SVM-NN, KNN-NN, and NB-NN, as classifiers to classify brain tumors.

## 3. Experimental Results and Discussions

All experiments were conducted in MATLAB using brain tumor images described in Section 2.1. Four pattern recognition neural network classifiers have been used. In addition, several statistical features such as GLDS, GLRLM, and GLCM (Table 1) were derived from different proposed scenarios (NS, SLT, and composite NS-SLT). The entire dataset was divided into training and testing sets with the ratio of 80 : 20 percent with the 10-fold cross-validation procedure. Performances of the three various scenarios were analyzed through a number of different measures [53, 54]. Further, performance evaluation accuracy of the statistical prediction system can also be done by calculating and analyzing the ROC curve. The ROC curve is a plot of the true-positive rate (sensitivity) versus the false-positive rate (1-specificity) for different thresholds over the entire range of each classifier output values. In contrast with the classification accuracies obtained from truth tables, ROC analysis is independent of class distribution or error costs.

**Figure 7:**
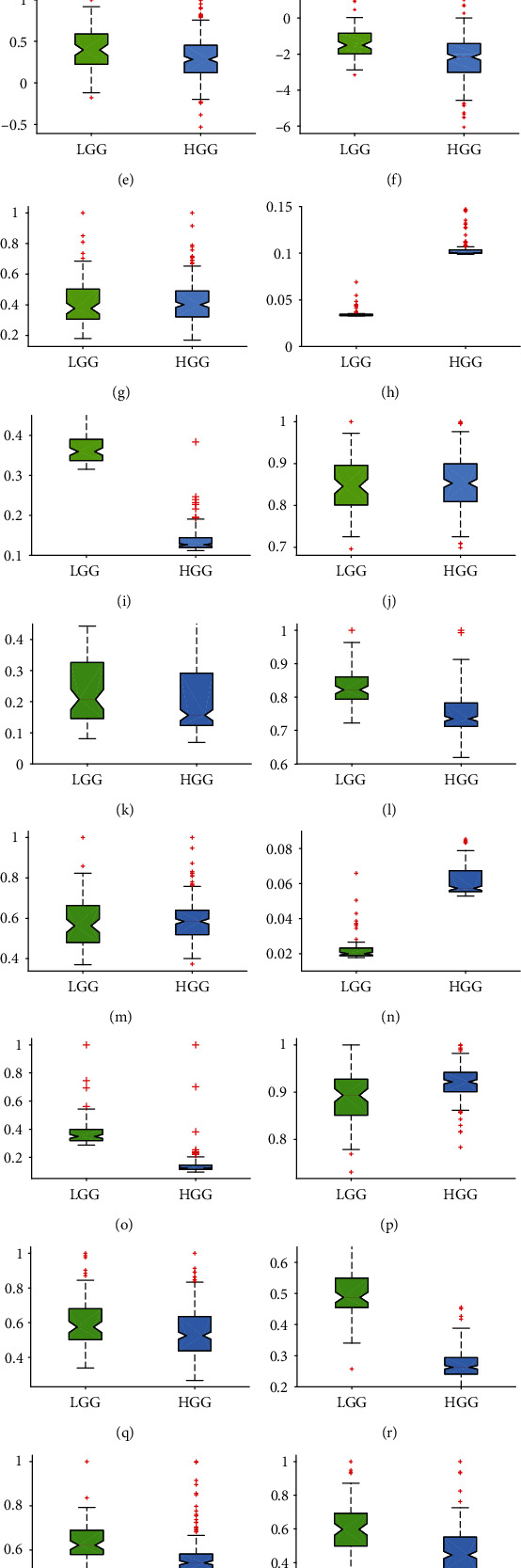
Boxplots of benign and malignant tumors: GLDS-ASM feature using (a) NS, (b) SLT, and (c) NS-SLT; GLDS mean feature using (d) NS, (e) SLT, and (f) NS-SLT; GLRLM-GLNU feature using (g) NS, (h) SLT, and (i) NS-SLT; GLRLM-SRE feature using (j) NS, (k) SLT, and (l) NS-SLT; GLRLM-RP feature using (m) NS, (n) SLT, and (o) NS-SLT; GLCM energy feature using (p) NS, (q) SLT, and (r) NS-SLT; and GLCM symmetry feature using (s) NS, (t) SLT, and (u) NS-SLT.

All results were first analyzed using boxplot diagrams that provided an overview of statistical values and distributions of benign and malignant brain tumors, as shown in Figure 7. Comparing sample medians regarding GLRLM-SRE (Figures 7(j)–7(l)), GLCM energy (Figures 7(p)–7(r)), and GLCM symmetry features (Figures 7(s)–7(u)), it is clearly visible that composite NS-SLT followed by texture feature extraction methods was significantly better compared to NS and SLT methods individually. Also, GLRLM-GLNU (Figures 7(g)–7(i)) and GLRLM-RP (Figures 7(m)–7(o)) features using both composite NS-SLT and SLT methods showed better performance than the NS-based texture method; however, GLDS-ASM and GLDS mean features (Figures 7(a)–7(f)) yield poor results, because an overlap of statistical features was observed between benign and malignant brain tumor categories in all scenarios. As a result, the composite NS-SLT method has an effective ability for brain tumor classification in comparison to other implemented techniques.

**Figure 8:**
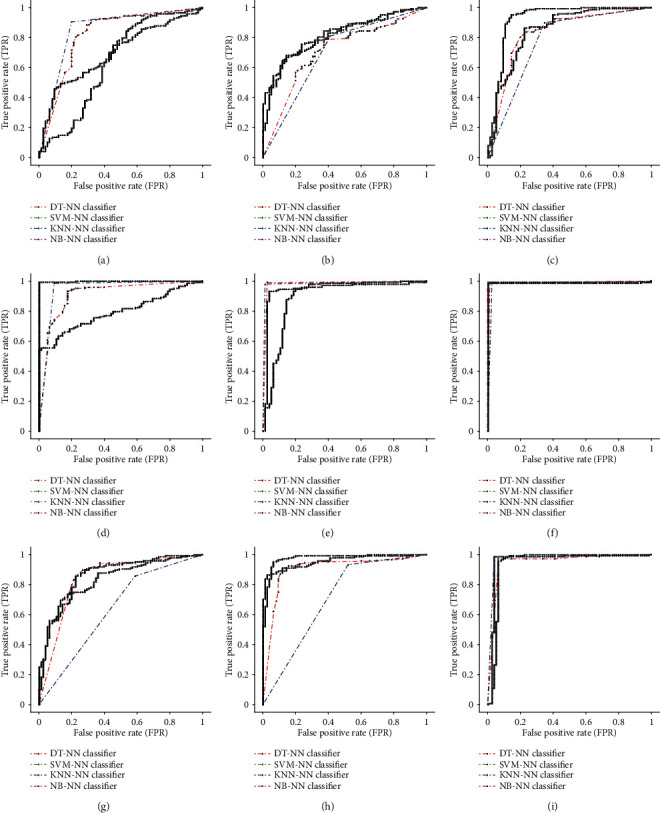
Comparison of ROC curves for GLDS, GLRLM, and GLCM features with various classifiers: ROC curve for GLDS features using (a) NS, (b) SLT, and (c) NS-SLT; ROC curve for GLRLM features using (d) NS, (e) SLT, and (f) NS-SLT; and ROC curve for GLCM features using (g) NS, (h) SLT, and (i) NS-SLT.

For each scenario, a different composition of each group of statistical and textural features was made. Table 2 presents the performance of each scenario followed by various pattern recognition classifiers (after applying ANOVA), starting by deriving each group (GLDS, GLRLM, and GLCM) features individually to see which group performs better in the classification stage with the minimum number of features. The performance metrics of NS, SLT, and composite NS-SLT scenarios for each of the proposed individual category of textural feature extraction corresponding to each scenario are shown in Table 3 and Figure 8. The GLRLM features derived by composite NS-SLT recorded the highest average classification accuracy rate with SVM-NN classifier 98.94% and an AUC of 0.99. As with all classifiers, GLRLM and GLCM features derived from composite NS-SLT achieved excellent average classification accuracy except for the GLDS features which achieved the lowest average classification results with KNN-NN and DT-NN classifiers, respectively.

**Figure 9:**
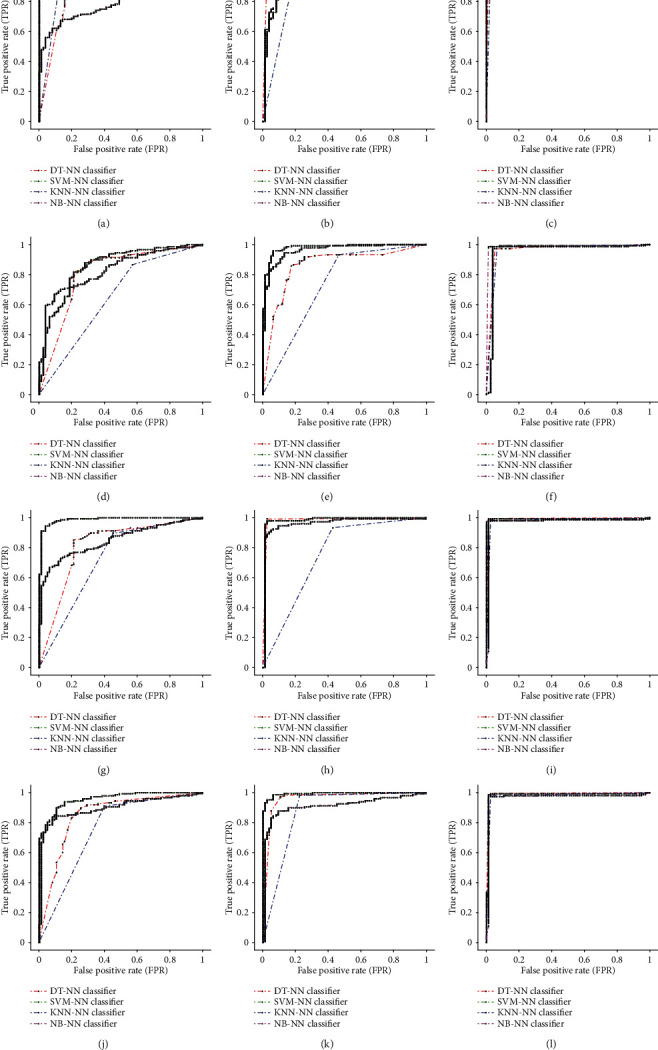
Comparison of ROC curves for different combinations of GLDS, GLRLM, and GLCM features with various classifiers: ROC curve for fusion of GLDS and GLRLM features using (a) NS, (b) SLT, and (c) NS-SLT; ROC curve for fusion of GLDS and GLCM features using (d) NS, (e) SLT, and (f) NS-SLT; ROC curve for fusion of GLRLM and GLCM features using (g) NS, (h) SLT, and (i) NS-SLT; and ROC curve for fusion of GLDS, GLRLM, and GLCM features using (j) NS, (k) SLT, and (l) NS-SLT.

This part of the results is concerned with showing the effect of combining texture features which are derived from NS, SLT, and composite NS-SLT techniques. The experimental results and comparison of ROC curves on fusion of texture features were mentioned in Table 4 and Figure 9. It was noticed that the classification performance using composite scenario yielded excellent results which go beyond NS or SLT techniques alone; also, the better precision and sensitivity parameters are achieved in most of the cases.

In all three scenarios, we also concluded that GLRLM features alone derived from the composite method gives superior results of 98.94% accuracy and an AUC of 0.99 with the SVM-NN classifier and by employing fewer number of features (only three features) whereas combining the GLRLM and GLDS together attains a highest prediction accuracy of 98.92% with an AUC of 0.99 whereas the classification accuracy of fused GLCM and GLDS features derived from NS was the lowest scoring 75.06% with an AUC of 0.64 with the KNN-NN classifier. Also, it is noticed that employing composite NS-SLT, NS, and SLT along with combining all the statistical texture features increases the overall accuracy in the case of the SVM-NN classifier but with the cost of employing 7, 10, and 10 features, respectively, and hence increasing system complexity.

**Figure 10:**
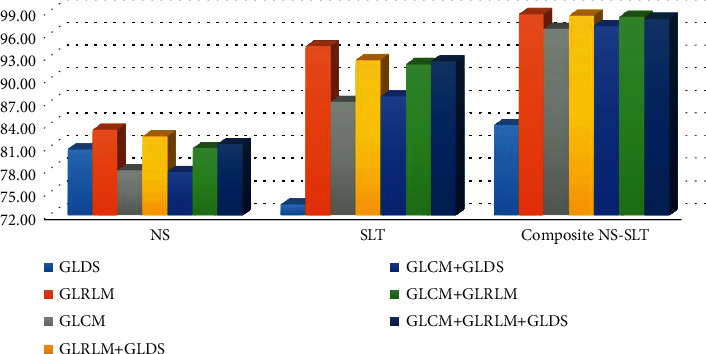
Comparison of average accuracies for individual and combined statistical features derived from SLT-NS, SLT, and NS.

**Figure 11:**
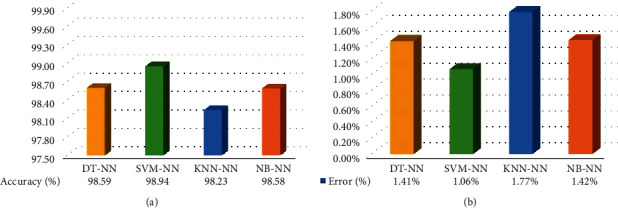
Performance of the proposed composite NS-SLT system with various classifiers: (a) accuracy and (b) error.

As a result of the comparison made between the proposed composite NS-SLT with NS and SLT methods, the GLRLM features derived from composite NS-SLT achieved best results, with a total average accuracy of 98.59% for all classifiers as shown in Figure 10 and the overall classification accuracies for the seven experiments conducted using composite NS-SLT which have been summarized in Table 5. Considering the obtained results, it is obvious that the proposed composite scenario outperforms others in both individual and combined statistical and textural features with various classifiers especially in the case of GLRLM features (Figure 11(a)). Moreover, in the proposed system, the error rate is less than 1.06%, 1.41%, 1.42%, and 1.77% with SVM-NN, DT-NN, NB-NN, and KNN-NN classifiers, respectively, as it is shown in Figure 11(b).

Finally, the performance of the proposed composite system is also compared with some existing state-of-the-art systems which used the same dataset and computing environment as shown in Table 6. The suggested system provides a promising result especially in terms of average classification accuracy when compared to existing methods. This is due to the integration carried out between SLT and neutrosophy which leads to gaining their advantages. However, the other researchers used some huge number of features while in the proposed system, only 3 features have been used with best performance results achieved.

From the above results, it is clear that the proposed system can successfully discriminate the tumor malignancy, which might help the doctors to make up a clear diagnosis based on their clinical expertise as well as the proposed tool as a second opinion.

## 4. Conclusion

Brain tumor MR image classification is a sophisticated process due to the variance and nonhomogeneity of tumors. Hence, the early identification of the tumor category (benign or malignant) is a critical issue that might save the life of patients. In this work, we have presented a novel automated brain tumor intelligent screening system using composite NS-SLT features extracted from the MR images. Based on research results and discussions, it is obviously concluded that the GLRLM features derived from composite NS-SLT are a promising technique to distinguish between malignant and benign brain tumors accurately on the available dataset. Our proposed architecture has achieved the highest prediction in terms of overall accuracy by 98.94%, precision of 0.96, sensitivity of 1.00, specificity of 0.98, and an AUC of 0.99 using the SVM-NN classifier (with just three relevant features) that are comparatively higher as compared with the state-of-the-art techniques. Furthermore, the recorded results have shown that our approach also achieves a high prediction performance of 98.59%, 98.58%, and 98.23% by using other (DT-NN, NB-NN, and KNN-NN) classifiers, respectively. In addition, using just three features reduces the complexity of the computation and enables fast and accurate decisions given to the doctors.

## Figures and Tables

**Table 1:** Statistical textural features extracted from dataset.

Technique	Textural features	No. of extracted features
GLCM	Homogeneity, energy, entropy, symmetry, contrast, correlation, moment 1, moment 2, moment 3, moment 4	10
CLRLM	Short run emphasis, long run emphasis, gray level nonuniformity, run percentage, run length nonuniformity, low gray level run emphasis, high gray level run emphasis	8
GLDS	Angular second moment, contrast, mean, entropy	4

**Table 2:** Comparison results of selected features with ANOVA from NS, SLT, and composite (NS-SLT).

Techniques	No. features	Feature selection method (ANOVA)					
		Scenario 1 (NS)		Scenario 2 (SLT)		Scenario 3 (NS-SLT)	
		No. features	*P* value	No. features	*P* value	No. features	*P* value
GLDS	4	2	3.43*E* − 08	2	5.54*E* − 06	2	4.27*E* − 58
GLRLM	8	3	1.43*E* − 53	3	2.87*E* − 33	3	3.12*E* − 44
GLCM	10	4	2.05*E* − 56	4	1.36*E* − 20	2	9.50*E* − 10
Fusion of GLRLM and GLDS	12	6	1.07*E* − 46	5	1.36*E* − 20	5	6.61*E* − 38
Fusion of GLCM and GLDS	14	7	4.31*E* − 31	5	4.51*E* − 24	6	9.15*E* − 04
Fusion of GLCM and GLRLM	18	9	1.74*E* − 53	8	1.36*E* − 20	5	9.50*E* − 10
Fusion of GLCM, GLRLM and GLDS	22	10	4.81*E* − 49	10	7.19*E* − 11	7	3.46*E* − 05

**Table 3:** Classification results obtained by GLDS, GLRLM, and GLCM features with various classifiers from NS, SLT, and composite NS-SLT methods, respectively. The highlighted accuracy in bold indicates the best classification result.

Features	Classifier methods	Techniques	Performance metrics				
			Accuracy (%)	Precision	Sensitivity	Specificity	AUC
GLDS	DT-NN	NS	85.61 ± 2.83	0.8 ± 0.100	0.68 ± 0.07	0.91 ± 0.03	0.81 ± 0.06
		SLT	71.40 ± 4.20	0.51 ± 0.09	0.48 ± 0.18	0.81 ± 0.06	0.70 ± 0.08
		NS-SLT	80.44 ± 5.35	0.67 ± 0.13	0.68 ± 0.16	0.85 ± 0.04	0.83 ± 0.06
	SVM-NN	NS	83.17 ± 3.22	0.97 ± 0.02	0.37 ± 0.12	1.00 ± 000	0.85 ± 0.10
		SLT	73.76 ± 1.76	0.72 ± 0.02	0.24 ± 0.05	0.94 ± 0.01	0.81 ± 0.01
		NS-SLT	81.18 ± 0.70	0.90 ± 0.03	0.41 ± 0.02	0.97 ± 0.01	0.85 ± 0.01
	KNN-NN	NS	87.70 ± 3.22	0.77 ± 0.05	0.79 ± 0.12	0.90 ± 0.02	0.85 ± 0.05
		SLT	74.53 ± 2.74	0.55 ± 0.08	0.59 ± 0.09	0.81 ± 0.02	0.69 ± 0.04
		NS-SLT	82.76 ± 2.15	0.74 ± 0.05	0.65 ± 0.05	0.90 ± 0.02	0.77 ± 0.03
	NB-NN	NS	76.08 ± 1.52	0.56 ± 0.12	0.36 ± 0.04	0.90 ± 0.01	0.72 ± 0.02
		SLT	74.15 ± 1.42	0.62 ± 0.11	0.35 ± 0.03	0.90 ± 0.01	0.81 ± 0.01
		NS-SLT	91.41 ± 1.74	0.93 ± 000	0.77 ± 0.04	0.97 ± 0.01	0.91 ± 0.02

GLRLM	DT-NN	NS	92.29 ± 2.29	0.87 ± 0.10	0.85 ± 0.06	0.94 ± 0.05	0.90 ± 0.05
		SLT	98.57 ± 0.71	0.97 ± 0.02	0.97 ± 0.01	0.99 ± 0.00	0.98 ± 0.00
		NS-SLT	98.59 ± 0.70	0.97 ± 0.02	0.97 ± 0.01	0.99 ± 0.00	0.98 ± 0.01
	SVM-NN	NS	89.84 ± 1.36	0.98 ± 0.01	0.62 ± 0.01	0.99 ± 0.00	0.98 ± 0.00
		SLT	90.13 ± 0.80	0.91 ± 0.03	0.71 ± 0.01	0.96 ± 0.01	0.89 ± 0.03
		NS-SLT	98.94 ± 0.02	0.96 ± 0.00	1.00 ± 0.00	0.98 ± 0.00	0.99 ± 0.00
	KNN-NN	NS	96.49 ± 1.04	0.96 ± 0.03	0.90 ± 0.05	0.98 ± 0.01	0.94 ± 0.03
		SLT	98.22 ± 0.04	0.95 ± 0.02	0.98 ± 0.01	0.98 ± 0.00	0.98 ± 0.01
		NS-SLT	98.23 ± 0.38	0.96 ± 0.01	0.97 ± 0.01	0.98 ± 0.00	0.98 ± 0.00
	NB-NN	NS	83.89 ± 2.81	0.78 ± 0.15	0.62 ± 0.06	0.91 ± 0.02	0.87 ± 0.01
		SLT	90.53 ± 3.16	0.88 ± 0.03	0.75 ± 0.12	0.96 ± 0.01	0.94 ± 0.02
		NS-SLT	98.58 ± 0.36	0.95 ± 0.01	1.00 ± 0.00	0.98 ± 0.00	0.98 ± 0.01

GLCM	DT-NN	NS	94.75 ± 1.60	0.92 ± 0.03	0.88 ± 0.04	0.97 ± 0.01	0.95 ± 0.02
		SLT	89.16 ± 2.09	0.78 ± 0.08	0.85 ± 0.10	0.90 ± 0.02	0.90 ± 0.05
		NS-SLT	96.10 ± 2.11	0.94 ± 0.02	0.93 ± 0.04	0.97 ± 0.02	0.95 ± 0.01
	SVM-NN	NS	93.37 ± 0.00	0.93 ± 0.00	0.80 ± 0.00	0.98 ± 0.00	0.98 ± 0.01
		SLT	90.53 ± 2.52	0.98 ± 0.01	0.65 ± 0.10	0.99 ± 0.00	0.97 ± 0.00
		NS-SLT	97.63 ± 0.36	0.97 ± 0.01	0.94 ± 0.01	0.98 ± 0.00	0.95 ± 0.01
	KNN-NN	NS	91.21 ± 3.54	0.86 ± 0.13	0.82 ± 0.08	0.94 ± 0.03	0.88 ± 0.03
		SLT	81.45 ± 1.77	0.78 ± 0.10	0.47 ± 0.07	0.93 ± 0.01	0.70 ± 0.03
		NS-SLT	97.65 ± 0.38	0.96 ± 0.00	0.95 ± 0.01	0.98 ± 0.00	0.97 ± 0.00
	NB-NN	NS	93.71 ± 1.39	0.90 ± 0.03	0.87 ± 0.05	0.96 ± 0.01	0.97 ± 0.01
		SLT	87.00 ± 2.48	0.81 ± 0.06	0.70 ± 0.04	0.92 ± 0.02	0.95 ± 0.00
		NS-SLT	95.29 ± 2.73	0.96 ± 0.00	0.88 ± 0.10	0.98 ± 0.00	0.94 ± 0.11

**Table 4:** Classification results obtained by different combinations of GLDS, GLRLM, and GLCM features with various classifiers from NS, SLT, and composite NS-SLT methods, respectively. The accuracy in bold indicates the best classification result.

Features	Classifier methods	Techniques	Performance metrics				
			Accuracy (%)	Precision	Sensitivity	Specificity	AUC
GLDS+GLRLM	DT-NN	NS	86.68 ± 4.95	0.76 ± 0.11	0.77 ± 0.08	0.90 ± 0.05	0.87 ± 0.06
		SLT	98.59 ± 0.35	0.97 ± 0.01	0.97 ± 0.00	0.99 ± 0.00	0.98 ± 0.00
		NS-SLT	98.23 ± 0.71	0.97 ± 0.02	0.97 ± 0.01	0.98 ± 0.00	0.98 ± 0.00
	SVM-NN	NS	84.23 ± 0.35	1.00 ± 0.00	0.40 ± 0.01	1.00 ± 0.00	0.99 ± 0.00
		SLT	90.55 ± 0.06	0.93 ± 0.04	0.69 ± 0.00	0.98 ± 0.00	0.94 ± 0.01
		NS-SLT	98.92 ± 0.03	0.97 ± 0.00	1.00 ± 0.00	0.98 ± 0.00	0.99 ± 0.00
	KNN-NN	NS	92.30 ± 1.78	0.91 ± 0.03	0.80 ± 0.05	0.96 ± 0.01	0.92 ± 0.02
		SLT	90.89 ± 2.06	0.85 ± 0.03	0.81 ± 0.06	0.94 ± 0.01	0.87 ± 0.03
		NS-SLT	97.88 ± 0.39	0.95 ± 0.00	0.97 ± 0.00	0.98 ± 0.00	0.97 ± 0.00
	NB-NN	NS	76.92 ± 1.73	0.59 ± 0.13	0.44 ± 0.04	0.88 ± 0.02	0.80 ± 0.02
		SLT	90.17 ± 1.77	0.84 ± 0.04	0.78 ± 0.02	0.94 ± 0.01	0.92 ± 0.01
		NS-SLT	98.57 ± 0.39	0.96 ± 0.00	1.00 ± 0.00	0.98 ± 0.00	0.98 ± 0.00

GLDS+GLCM	DT-NN	NS	83.14 ± 3.80	0.71 ± 0.07	0.64 ± 0.07	0.89 ± 0.04	0.80 ± 0.05
		SLT	85.61 ± 3.52	0.73 ± 0.08	0.76 ± 0.07	0.89 ± 0.02	0.86 ± 0.07
		NS-SLT	96.04 ± 2.08	0.94 ± 0.04	0.92 ± 0.06	0.97 ± 0.01	0.96 ± 0.01
	SVM-NN	NS	83.17 ± 0.00	0.97 ± 0.00	0.37 ± 0.00	0.99 ± 0.00	0.85 ± 0.01
		SLT	91.92 ± 1.40	0.97 ± 0.01	0.71 ± 0.05	0.99 ± 0.00	0.97 ± 0.00
		NS-SLT	97.64 ± 0.39	0.97 ± 0.00	0.94 ± 0.01	0.98 ± 0.00	0.95 ± 0.02
	KNN-NN	NS	75.06 ± 2.47	0.54 ± 0.07	0.42 ± 0.08	0.86 ± 0.03	0.64 ± 0.04
		SLT	82.84 ± 2.45	0.77 ± 0.12	0.53 ± 0.04	0.93 ± 0.03	0.73 ± 0.02
		NS-SLT	96.86 ± 1.41	0.96 ± 0.02	0.93 ± 0.04	0.98 ± 0.00	0.95 ± 0.02
	NB-NN	NS	79.29 ± 1.42	0.62 ± 0.08	0.51 ± 0.04	0.89 ± 0.02	0.83 ± 0.01
		SLT	90.54 ± 1.72	0.87 ± 0.05	0.77 ± 0.03	0.95 ± 0.02	0.96 ± 0.00
		NS-SLT	97.63 ± 1.74	0.97 ± 0.00	0.94 ± 0.02	0.98 ± 0.00	0.98 ± 0.01

GLRLM+GLCM	DT-NN	NS	81.45 ± 6.72	0.66 ± 0.15	0.68 ± 0.14	0.86 ± 0.05	0.81 ± 0.08
		SLT	98.58 ± 0.36	0.97 ± 0.01	0.97 ± 0.00	0.99 ± 0.00	0.98 ± 0.00
		NS-SLT	98.59 ± 1.39	0.98 ± 0.01	0.95 ± 0.03	0.99 ± 0.00	0.99 ± 0.00
	SVM-NN	NS	90.54 ± 1.81	0.96 ± 0.00	0.66 ± 0.07	0.99 ± 0.00	0.98 ± 0.00
		SLT	93.31 ± 1.07	0.93 ± 0.02	0.79 ± 0.04	0.98 ± 0.00	0.98 ± 0.00
		NS-SLT	98.60 ± 0.72	0.96 ± 0.02	0.98 ± 0.00	0.98 ± 0.00	0.99 ± 0.00
	KNN-NN	NS	83.87 ± 1.81	0.74 ± 0.09	0.61 ± 0.03	0.91 ± 0.02	0.72 ± 0.02
		SLT	83.84 ± 1.07	0.78 ± 0.07	0.57 ± 0.04	0.93 ± 0.01	0.75 ± 0.01
		NS-SLT	97.90 ± 0.37	0.95 ± 0.00	0.97 ± 0.01	0.98 ± 0.00	0.97 ± 0.00
	NB-NN	NS	81.76 ± 1.46	0.68 ± 0.04	0.64 ± 0.04	0.88 ± 0.01	0.84 ± 0.00
		SLT	92.29 ± 1.41	0.87 ± 0.04	0.84 ± 0.04	0.95 ± 0.01	0.96 ± 0.01
		NS-SLT	97.89 ± 1.04	0.95 ± 0.01	0.97 ± 0.01	0.98 ± 0.00	0.97 ± 0.02

GLDS+GLRLM+GLCM	DT-NN	NS	86.34 ± 6.71	0.79 ± 0.14	0.7 ± 0.16	0.91 ± 0.05	0.85 ± 0.04
		SLT	95.07 ± 3.15	0.93 ± 0.05	0.86 ± 0.08	0.98 ± 0.01	0.95 ± 0.04
		NS-SLT	98.22 ± 0.72	0.98 ± 0.00	0.94 ± 0.03	0.99 ± 0.00	0.99 ± 0.00
	SVM-NN	NS	95.77 ± 1.07	0.98 ± 0.00	0.85 ± 0.04	0.99 ± 0.00	0.94 ± 0.00
		SLT	95.43 ± 0.72	0.95 ± 0.01	0.86 ± 0.03	0.98 ± 0.00	0.99 ± 0.00
		NS-SLT	98.23 ± 0.73	0.95 ± 0.02	0.98 ± 0.00	0.98 ± 0.00	0.98 ± 0.00
	KNN-NN	NS	82.82 ± 2.81	0.71 ± 0.09	0.60 ± 0.09	0.91 ± 0.03	0.75 ± 0.04
		SLT	92.61 ± 1.82	0.94 ± 0.04	0.77 ± 0.06	0.98 ± 0.01	0.87 ± 0.03
		NS-SLT	97.89 ± 0.37	0.95 ± 0.01	0.97 ± 0.00	0.98 ± 0.00	0.97 ± 0.00
	NB-NN	NS	81.71 ± 3.16	0.65 ± 0.11	0.59 ± 0.04	0.89 ± 0.02	0.90 ± 0.01
		SLT	86.33 ± 8.42	0.85 ± 0.11	0.73 ± 0.13	0.90 ± 0.08	0.91 ± 0.05
		NS-SLT	97.52 ± 1.07	0.93 ± 0.02	0.98 ± 0.00	0.97 ± 0.01	0.96 ± 0.02

**Table 5:** Classification results for individual and combined texture features derived from SLT in the neutrosophic domain (composite NS-SLT). The accuracies in bold indicate the best classification result.

Statistical features	Classifier method				
	DT-NN (%)	SVM-NN (%)	KNN-NN (%)	NB-NN (%)	Average accuracy (%)
GLDS	80.44	81.18	82.76	91.41	83.95
GLRLM	**98.59**	**98.94**	**98.23**	**98.58**	**98.59**
GLCM	96.10	97.63	97.65	95.29	96.67
Fusion of GLRLM and GLDS	98.23	98.92	97.88	98.57	98.40
Fusion of GLCM and GLDS	96.04	97.64	96.86	97.63	97.04
Fusion of GLCM and GLRLM	98.59	98.60	97.90	97.89	98.25
Fusion of GLCM, GLRLM, and GLDS	98.22	98.23	97.89	97.52	97.97

**Table 6:** Comparison of proposed classification accuracy with recent techniques.

Author	Year	Techniques used on the same BraTS17 dataset		Classification accuracy (%)
		Feature extraction	Classifier	
Banerjee et al. [10]	2017	ConvNet model	DCNN	97.19
Cho et al. [51]	2018	Radiomic approach (ISZM, GLCM, SFB, and HBF)	Logistic, SVM, and RF	92.92
Sharif et al. [9]	2019	Scattering transform, wavelet transform, and local Gabor binary pattern	HCS-DBN	94.50
Raju et al. [55]	2019	SFTA and LBP	MSVM	96.90
Proposed work		GLRLM—composite NS-SLT	SVM-NN, DT-NN, KNN-NN, and NB-NN	98.94

## Data Availability

The dataset used to support the findings of this study is from the MICCAI BraTS Challenge 2017 (https://www.med.upenn.edu/sbia/brats2017/data.html).
